# Preoperative Fasting Protects against Renal Ischemia-Reperfusion Injury in Aged and Overweight Mice

**DOI:** 10.1371/journal.pone.0100853

**Published:** 2014-06-24

**Authors:** Franny Jongbloed, Ron W. F. de Bruin, Jeroen L. A. Pennings, César Payán-Gómez, Sandra van den Engel, Conny T. van Oostrom, Alain de Bruin, Jan H. J. Hoeijmakers, Harry van Steeg, Jan N. M. IJzermans, Martijn E. T. Dollé

**Affiliations:** 1 Department of Surgery, Laboratory for Experimental Transplantation and Intestinal Surgery (LETIS), Erasmus University Medical Center, Rotterdam, The Netherlands; 2 Laboratory of Health Protection Research, National Institute of Public Health and the Environment, Bilthoven, The Netherlands; 3 Department of Genetics, Erasmus University Medical Center, Rotterdam, The Netherlands; 4 Facultad de Ciencias Naturales y Matemáticas, Universidad del Rosario, Bogotá, Colombia; 5 Dutch Molecular Pathology Center, Department of Pathobiology Faculty of Veterinary Medicine, Utrecht University, Utrecht, The Netherlands; 6 Department of Toxicogenetics, Leiden University Medical Center, Leiden, The Netherlands; The University of Manchester, United Kingdom

## Abstract

Ischemia-reperfusion injury (IRI) is inevitable during kidney transplantation leading to oxidative stress and inflammation. We previously reported that preoperative fasting in young-lean male mice protects against IRI. Since patients are generally of older age with morbidities possibly leading to a different response to fasting, we investigated the effects of preoperative fasting on renal IRI in aged-overweight male and female mice. Male and female F1-FVB/C57BL6-hybrid mice, average age 73 weeks weighing 47.2 grams, were randomized to preoperative ad libitum feeding or 3 days fasting, followed by renal IRI. Body weight, kidney function and survival of the animals were monitored until day 28 postoperatively. Kidney histopathology was scored for all animals and gene expression profiles after fasting were analyzed in kidneys of young and aged male mice. Preoperative fasting significantly improved survival after renal IRI in both sexes compared with normal fed mice. Fasted groups had a better kidney function shown by lower serum urea levels after renal IRI. Histopathology showed less acute tubular necrosis and more regeneration in kidneys from fasted mice. A mRNA analysis indicated the involvement of metabolic processes including fatty acid oxidation and retinol metabolism, and the NRF2-mediated stress response. Similar to young-lean, healthy male mice, preoperative fasting protects against renal IRI in aged-overweight mice of both genders. These findings suggest a general protective response of fasting against renal IRI regardless of age, gender, body weight and genetic background. Therefore, fasting could be a non-invasive intervention inducing increased oxidative stress resistance in older and overweight patients as well.

## Introduction

The rate of morbidity and mortality in patients with end-stage renal disease (ESRD) is greatly decreased by kidney transplantation, although it brings along its own set of complications like delayed graft function (DGF) and rejection [1]. DGF, defined as the need of dialysis in the first week after transplantation, occurs in almost 25% of all kidney transplantations in the USA [2]. Because elderly patients also benefit from a renal transplant, the number of transplantations performed in these patients has increased [3]. With the already existing shortage of donors, this has widened the gap between organ availability and demand. In an attempt to overcome this shortage, guidelines for extended donation are developed [4]. These extended criteria donors (ECD) are generally of older age, with existing morbidities like obesity and diabetes mellitus [5]. ECD kidneys show higher risks of DGF, acute rejection and graft failure, with recipient and donor age as independent risk factors [6], [7]. Hence, practices improving transplantation success rates are urgently needed.

Ischemia-reperfusion injury (IRI) is an unavoidable consequence of organ transplantation leading to a deleterious activation of cellular oxidases causing oxidative damage, tissue injury and inflammation [8], [9]. IRI has been identified as a major risk factor for the development of DGF and rejection, with ECD kidneys being even more vulnerable to ischemic damage [10]. A potentially protective intervention against IRI is dietary restriction (DR), a short-term reduction of caloric intake before induction of ischemia. Long-term DR has been shown to prolong both health- and lifespan [11], [12]. Although its mechanisms have not yet been elucidated, increased resistance to oxidative stress is likely to be involved. In addition, short-term DR and fasting as a more acute approach of DR have been found to increase resistance against oxidative damage as well, including IRI [9], [13]. Previously, we have shown that both three days of fasting and two weeks of 30% DR induce robust protection against renal IRI in mice [9], [14], [15]. However, these studies have been conducted with healthy young-lean male mice. The relevance of these findings for a heterogeneous group of patients which are generally older and suffer from disease is uncertain since the response induced by fasting and DR under these conditions is unknown [16], [17]. Therefore, the aim of this study is to examine the effect of age, body weight, gender, and genetic background on the protection against renal IRI induced by fasting. We show that preoperative fasting protects against the effects of IRI in aged-overweight male and female mice comparable to young-lean mice. Subsequently, we compared gene expression profiles of kidneys of young-lean mice with those of aged-overweight mice before and after fasting and identified several metabolic processes including fatty acid oxidation and retinol metabolism as well as pathways involved in the oxidative stress response as likely candidates involved in the induction of increased stress resistance by fasting.

## Results

### Three-day fasting confers resistance to renal ischemia in aged-overweight mice

**Figure 1:**
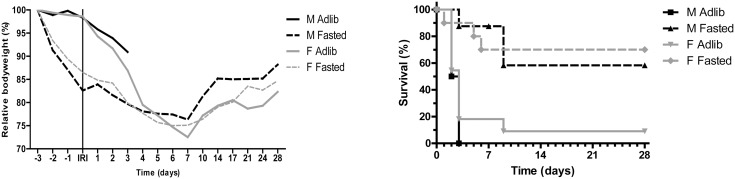
Relative body weight and survival. Relative body weight (A) and survival (B) of male and female mice undergoing 37 and 60 minutes of renal ischemia-reperfusion injury (IRI), respectively, preceded by 3 day fasting or ad libitum feeding. In the first week after surgery, both fasted groups gradually lost weight after which they slowly gained weight in the weeks thereafter. Both fasted groups show a significantly improved survival: p = 0.0171 for males, p = 0.0040 for females. M = male, F = female, Adlib = ad libitum fed.

Male and female F1-FVB/C57BL6-hybrid mice were either fasted for three days by permitting water only or fed ad libitum preceding surgery. The mean baseline weight was 47.4±5.1 and 48.0±7.6 grams for male and female mice, respectively, demonstrating their overweight phenotype acquired by ad libitum conditions compared to their mean body weight at 12 weeks: 31.4±3.1 (male) and 24.2±2.4 (female) (data not shown). Fasting resulted in a net body weight loss of 8.2±0.5 and 6.5±0.9 grams in males and females, respectively, correlating with approximately 17% and 14% of their body weight before fasting (Figure 1A). At surgery, bilateral renal ischemia was applied for 37 minutes in male and 60 minutes in female mice followed by reperfusion. Both male and female fasted mice had a significant improved survival compared to the ad libitum fed groups (Figure 1B). All 8 ad libitum male mice died or were sacrificed because of morbidity indicative of kidney failure within three days following reperfusion, whereas 7 out of 8 fasted males survived through day 7. At day 7, four male mice were sacrificed for pathological analysis and censored in Figure 1B. Two of the three remaining mice survived in good health through day 28. In the fasted female group, one mouse was found dead on day 1 after IRI because of secondary causes (sutures removed by animal) and was excluded from further analyses. One of the 11 ad libitum fed females survived the observation period, while the majority, nine in total, died or were sacrificed due to morbidity within 3 days following IRI. At 28 days, 7 of 10 fasted female mice were still alive and appeared healthy. All fasted mice that died showed indications of irreversible kidney failure (morbidity and loss of kidney function, see serum and histology data described further on).

### Preoperative fasting leads to improved kidney function and recovery after IRI

**Figure 2:**
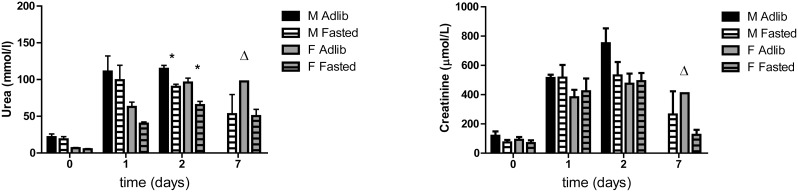
Kidney function after renal ischemia-reperfusion injury. Kidney function of male and female mice after undergoing 37 and 60(A) Serum urea levels are significantly lower in fasted males and females on day 2 after renal IRI. (B) Serum creatinine levels show no significant differences between fasted and ad libitum fed mice. M = male, F = female, Adlib = ad libitum fed. Δ = no standard deviation is shown as only two animals comprised this group.

**Figure 3:**
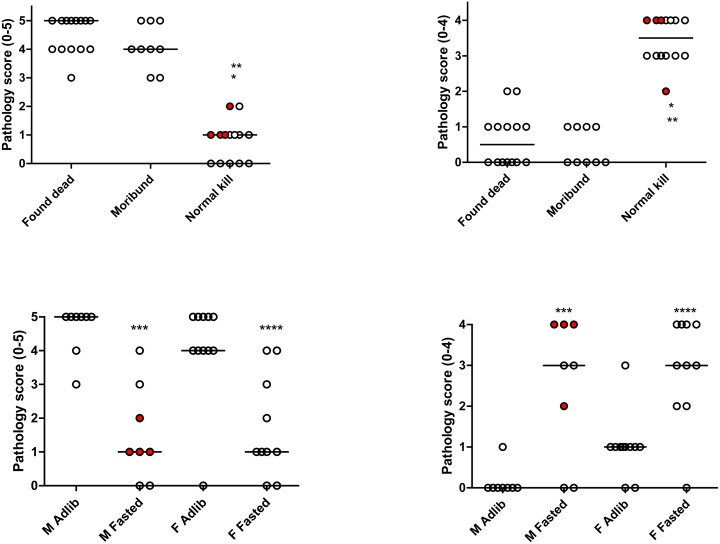
Histopathological analysis of kidneys after renal ischemia-reperfusion injury. Histopathological analysis of kidneys of male and female mice after IRI. Mice sacrificed at the end of the experiment showed significantly less acute tubular necrosis (A) and significantly more tubular epithelial regeneration (B). Divided by intervention, both male and female fasted mice also showed less necrosis (C) and more regeneration (D). Fasted male mice sacrificed at day 7 showed similar pathology scores as the fasted mice at day 28 (mice indicated by the red symbols).*  =  significance (p<0.05) compared to the group ‘Found dead’, **  =  compared to the group ‘Moribund’, ***  =  compared to ad libitum fed males, ****  =  compared to ad libitum fed females.

**Figure 4:**
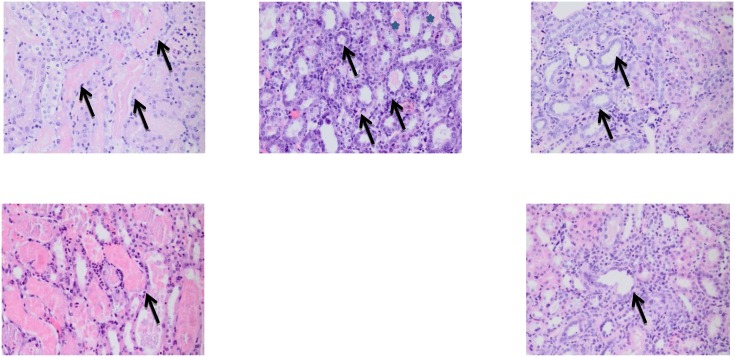
Histological images of kidneys with acute tubular necrosis and tubular epithelial regeneration. Representative images of HE stained kidney sections with acute tubular necrosis or tubular epithelial regeneration. (A) Multifocal severe acute tubular necrosis with typical diluted tubules, flattened epithelial lining and granular casts inside the tubules (arrows) in an ad libitum fed male mouse 4 days after 37 minutes of IRI. (B) On day 7, male fasted mice already show a high degree of regeneration (arrows) with minimal necrosis (stars). (C) Multifocal tubular regeneration as shown by mitosis bodies along the epithelial line (arrows) in a fasted male mouse 28 days after 37 minutes of IRI. (D) Multifocal severe acute tubular necrosis in ad libitum fed female mouse 4 days after 60 minutes of IRI. (E) Multifocal tubular regeneration in a fasted female mouse 28 days after 60 minutes of IRI.

To measure the effect of fasting on kidney function after IRI, serum urea and creatinine levels were measured at days 0, 1 and if applicable, 2 and 7 after surgery. Compared to ad libitum fed mice, serum urea levels in male and female fasted aged mice were lower on postoperative day one, which became significant on day 2 (males p = 0.001; females p = 0.016; Figure 2A). Significance could not be determined on day 7 post-IRI as only 2 ad libitum females remained. Serum creatinine failed to show statistically significant differences between groups (Figure 2B). Kidney damage after IRI was further assessed by histopathology. After sorting by cause of death, mice found dead and mice that were moribund and sacrificed showed severe acute tubular necrosis (Figure 3A), a major determinant of renal IRI. Median tubular necrosis scores in these groups, with 0 indicating no and 5 severe necrosis, were 5 and 4, respectively, with no significant differences between the two groups, demonstrating that moribund mice indeed suffered from severe kidney failure independent of the day histology was determined. In contrast, the surviving mice had a median pathology score of 1 (p<0.001) (Figure 3A). As time after IRI might influence the severity of the pathological changes, 4 fasted male mice were sacrificed at day 7 and histology was compared with mice that died due to kidney failure. The tubular necrosis score of these 4 sacrificed mice was identical to that of the mice sacrificed at day 28 (median score 1), showing a similar contrast with the mice that died due to kidney failure, indicative of an acute protective effect of the diet. The amount of tubular regeneration is scored between 0 and 4 with 4 as the strongest regenerative response. Mice found dead or moribund scored a median pathology score for tubular regeneration of 0.5 and 0, respectively, and the fasted mice had a median score of 3.5, including mice sacrificed at day 7 and at later time-points (p<0.0001) (Figure 3B). When subdivided by gender and diet group, fasted male and female mice showed significantly less necrosis (p = 0.0018 and p = 0.0035, respectively) and significantly more regeneration (p = 0.0089 and 0.0036, respectively) compared to the ad libitum fed groups (Figures 3 C/D). Representative images of the pathological lesions observed in the different groups are shown in Figure 4.

### Fasted aged-overweight mice show a gradual weight gain after renal IRI

After IRI, the fasted male and female mice showed a 5% and 10% weight loss, respectively, in the first 5 postoperative days, after which their weight stabilized (Figure 1A). From day 7 on, they gradually gained weight and eventually reached their preoperative weight (t = 0) at day 28. In contrast, the ad libitum fed mice showed a larger and faster weight loss after IRI. The ad libitum fed males lost 7% in three days, after which they had all died. The ad libitum fed females lost slightly more relative weight during the same period (10%). By day 5, they had lost 21% of their weight, resulting in a total weight slightly below that reached by the fasted animals. After day 7, the single surviving ad libitum fed female mouse showed a similar trend in body weight gain as the fasted mice.

### Gene expression profiles in kidneys show overlap between young and aged male mice in response to fasting

**Figure 5:**
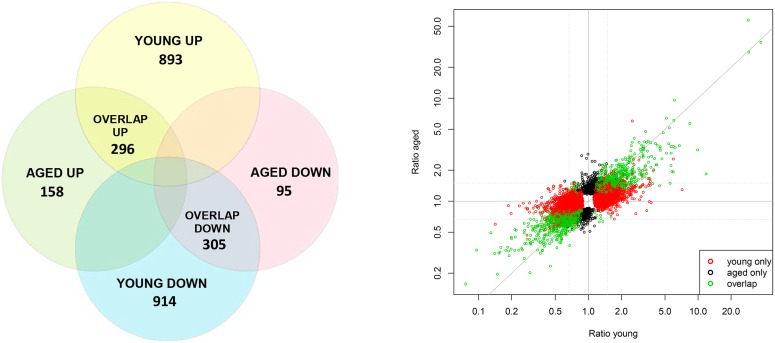
Venn diagram and scatterplot of microarray data after fasting in young and aged mice. A) Venn diagram of the significantly differentially up- and down-regulated (FDR ≤5%, FC ≥1.5) and overlapping probe sets in kidneys of three days fasted aged and young mice in comparison with normal fed control mice. B) Scatter plot comparing up- and down-regulated trends and their fold ratios of probe sets significantly regulated (FDR ≤5%) in young-lean (red), aged-overweight mice (black) or overlapping in both groups (green). Without fold change cut-off, 85.0% of the genes showed the same directionality in both age groups. The gray solid line represents the reference diagonal (ratio young  =  ratio aged); the gray dotted lines show the 1.5 fold change cutoffs applied in 5A.

Firstly, gene expression profiles were compared between kidneys of ad libitum fed and three days fasted aged male mice (without renal IRI) by whole-genome mRNA microarray analysis. The total number of probe sets differentially regulated was assessed. In total, 854 out of 45141 probe sets were significantly differentially regulated (FDR ≤5%) in the fasted aged mice with a fold change ≥1.5. Of these 854 probe sets, 454 probe sets were up-regulated and 400 down-regulated (Figure 5A). Secondly, since fasting was shown to confer resistance to IRI in young-lean male mice [9], we performed a similar mRNA analysis in kidneys from young mice. A total of 2408 probe sets were found to be significantly differentially regulated in fasted compared with ad libitum fed young mice with 1189 up-regulated and 1219 down-regulated probe sets (Figure 5A). The Venn diagram in Figure 5A shows that most probe sets (70%) found in aged mice overlap with the probe sets found in young mice. Young mice show an almost three-fold increase of significantly regulated genes indicative of a stronger, more extensive response to fasting in young compared to aged mice. To verify whether gene expression changes followed the same directionality, the fold expression changes of all significant probe sets (FDR ≤5%) in young or aged mice were plotted against each other (Figure 5B). The majority of these probe sets (85.0%) had similar directional changes in kidneys of young and aged mice, although the fold changes of the aged kidneys were generally lower compared to young.

To determine the effect on a biological level, functional annotation and pathway overrepresentation analyses of probe sets with FDR ≤5% and FC ≥1.5, were performed with Ingenuity software. The top up- and down-regulated genes (FC ≥5) with corresponding p-values resulting from the functional annotation are provided as supplementary tables by age group (Table S1-S4 in File S1). Pathway analysis in aged mice resulted in the highest overrepresented pathways being *LXR/RXR Activation, Cholesterol Biosynthesis* and *Fatty Acid β-Oxidation* (Table 1). The most overrepresented pathways in young-lean male mice were *Cholesterol Biosynthesis*, *NRF2-mediated Oxidative Stress Response* and *LXR/RXR Activation*. Amongst the top 15 regulated pathways, 6 pathways are represented in both aged and young mice (Table 1), demonstrating the similarity of the individual pathway results.

**Table 1:** Top 15 canonical pathways in kidney of aged and young mice in response to fasting compared with ad libitum.

Canonical Pathways AGED	P-value	Genes Ratio
LXR/RXR Activation	9.68E-08	17/139 (12.2%)
Fatty Acid β-oxidation I	1.30E-07	9/45 (20.0%)
Superpathway of Cholesterol Biosynthesis	1.30E-07	9/87 (10.3%)
LPS/IL-1 Mediated Inhibition of RXR Function	6.08E-07	22/245 (9.0%)
Stereate Biosynthesis I (Animals)	6.32E-06	8/49 (16.3%)
Cholesterol Biosynthesis I/II/III	2.31E-05	5/40 (12.5%)
TR/RXR Activation	1.96E-04	10/109 (9.2%)
NRF2-mediated Oxidative Stress Response	2.96E-04	15/195 (7.7%)
Dopamine Degradation	5.84E-04	5/38 (13.2%)
Androgen Biosynthesis	5.91E-04	4/26 (15.4%)
Mevalonate Pathway I	7.87E-04	4/29 (13.8%)
Alanine Degradation III/Biosynthesis II	8.86E-04	2/6 (33.3%)
γ-linolenate Biosynthesis II (Animals)	1.31E-03	4/24 (16.7%)
Superpathway of Geranylgeranyldiphosphate Biosynthesis I (via Mevalonate)	2.04E-03	4/37 (10.8%)

Canonical Pathways YOUNG	P-value	Genes Ratio
Superpathway of Cholesterol Biosynthesis	1.19E-08	14/87 (16.1%)
NRF2-mediated Oxidative Stress Response	3.46E-07	35/195 (17.9%)
LXR/RXR Activation	4.46E-07	27/139 (19.4%)
Cholesterol Biosynthesis I/II/III	1.16E-06	8/40 (20.0%)
LPS/IL-1 Mediated Inhibition of RXR Function	6.87E-06	37/245 (15.1%)
VDR/RXR Activation	8.03E-05	17/88 (19.3%)
Acute Phase Response Signaling	1.04E-04	28/181 (15.5%)
Glucocorticoid Receptor Signaling	1.24E-04	38/299 (12.7%)
Xenobiotic Metabolism Signaling	2.70E-04	38/288 (13.2%)
AMPK Signaling	2.75E-04	23/181 (12.7%)
Fatty Acid β-oxidation I	3.17E-04	9/45 (20.0%)
GADD45 Signaling	5.23E-04	7/24 (29.2%)
PPARα/RXRα Activation	5.39E-04	27/200 (13.5%)
Production of Nitric Oxide and Reactive Oxygen Species	6.44E-04	27/212 (12.7%)
FXR/RXR Activation	8.48E-04	16/110 (14.5%)

Canonical pathways differentially regulated by 3 days fasting in kidneys from (A) aged mice and (B) young mice compared to their ad libitum controls. Pathways are ranked by their corresponding p-value. Genes Ratio is the amount of genes significantly differentially regulated in the pathway.

The overlapping probe sets in both young and aged mice presumably contain those in the kidney that contribute to resistance to IRI. Therefore, the pathway overrepresentation was repeated for the 601 probe sets significantly expressed in both age groups (Table 2). The top overrepresented pathways involve RXR Activation, Fatty Acid β-oxidation or NRF2-mediated stress response, partly overlapping with the age group specific pathway analyses.

**Table 2:** Top 15 canonical pathways overlapping in aged and young mice in response to fasting compared with ad libitum.

Canonical Pathways OVERLAP	P-value	Genes Ratio
LPS/IL-1 Mediated Inhibition of RXR Function	7.85E-07	18/245 (7.3%)
LXR/RXR Activation	1.27E-06	13/139 (9.4%)
Fatty Acid β-oxidation I	2.00E-06	7/45 (15.6%)
Superpathway of Cholesterol Biosynthesis	2.85E-05	6/87 (6.9%)
Dopamine Degradation	1.10E-04	5/38 (13.2%)
Androgen Biosynthesis	1.50E-04	4/26 (15.4%)
Alanine Degradation III/Biosynthesis II	4.22E-04	2/6 (33.3%)
Retinoate Biosynthesis I	4.57E-04	5/37 (13.5%)
Stereate Biosynthesis I (Animals)	7.01E-04	5/49 (10.2%)
NRF2-mediated Oxidative Stress Response	1.29E-03	11/195 (5.6%)
Serotonin Degradation	1.56E-03	6/78 (7.8%)
TR/RXR Activation	1.84E-03	7/109 (6.4%)
FXR/RXR Activation	1.97E-03	7/110 (6.4%)
Cholesterol Biosynthesis I	2.12E-03	3/40 (7.5%)
Bile Acid Biosynthesis, Neutral Pathway	2.12E-03	3/58 (5.2%)

Canonical pathways overlapping in aged and young mice after 3 days of fasting with their corresponding geometric mean p-value and the percentage of regulated genes.

Because of their relatively high fold change and their involvement in processes regulated by fasting found in the analysis, *Pparα*, *Gst2*, *Cyp4a14* and *Sc4mol* were validated by qRT-PCR. The qRT-PCR data confirmed the up-regulation of *Pparα*, *Gst2*, *Cyp4a14* with fold increases of 2.5, 8.0 and 106 respectively in young and 2.2, 3.4 and 147, respectively in aged kidneys. The down-regulation of *Sc4mol* was also validated with qRT-PCR and showed a significant 1.77-fold decrease in young and 1.72-fold in aged kidneys (Table 3).

**Table 3:** Validation via qRT-PCR of four genes found to be significant in the array analysis.

Gene	Aged (Affy)	Aged – qPCR	Young (Affy)	Young – qPCR
*Pparα*	2.53/3.00e-09	2.5/6.83e-07	2.74/1.45e-05	2.2/0.0005
*Gsta2*	5.52/2.45e-08	8.0/3.12e-05	8.72/3.73e-05	3.4/1.52e-05
*Cyp4a14*	344.47/4.11e-09	106/6.16e-06	126.72/9.85e-07	147/0.0003
*Sc4mol*	−2.64/5.16e-07	−1.77/0.005	−4.98/1.69e-06	−1.72/0.007

Fold ratios and corresponding p-values obtained via microarray analysis (Affy) and qRT-PCR (qPCR) of four genes found to be significant in the performed array analysis.

## Discussion

With publications dating back 80 years, dietary restriction is one of the most extensively investigated interventions in biology [18], [19]. Long-term DR leads to prolongation of health- and lifespan in many animal species [11] as well as an increase in acute stress resistance [20]. Induction of organ ischemia is a widely used model to examine the effects of DR, since it has been shown to increase the resistance against ischemic injury in organs like heart and brain [21], [22]. Previous research done in one of our laboratories showed that applying short periods of DR (2 and 4 weeks 30% DR) as well as 3 days of fasting, conveyed strong protection against morbidity and mortality induced by IRI in the kidney as well as decreased morbidity and improved liver function in a non-lethal liver IRI model [9], [23], [24]. A drawback of the majority of reports is that for practical reasons the same animal model was utilized, namely healthy young (8–12 weeks) male mice of one genetic background (C57BL/6). These mice poorly represent the heterogeneous human population. In particular, this is valid for ECD which are older and usually overweight. In this study, we showed that preoperative fasting strongly protects both male and female aged-overweight mice against renal IRI, with a significantly reduced mortality after IRI and better preserved kidney function and morphology. Compared to young mice, the recovery of body weight in fasted aged mice takes longer, probably at least in part due to their greater fat reserve and therefore reduced need to restore body weight in order to survive. Previously we observed that fasted young mice started eating shortly after surgery and showed a rapid increase in body weight. We showed that postoperative food intake and associated weight gain did not contribute to the observed protection [9]. The prolonged modest postoperative weight loss in the fasted aged mice provides additional evidence that postoperative feeding is not involved. In addition, the relative body weight loss in aged mice was less than in the young cohort, leaving these mice with more remaining body fat after fasting and IRI. These results indicate that weight loss and reduction of body fat coincide with but do not contribute to the protection against IRI induced by preoperative deprivation of food.

We compared and analyzed the overlap of gene expression data of young and aged mice via microarray analysis, narrowing down genes and pathways possibly involved in the effect of fasting. We found a striking corresponding directionality in overlapping genes of both groups with the changes being more pronounced and more often significant in the young group. Possibly, aged organs and tissues are not able to mount a more vigorous response compared to young animals. Alternatively or in addition, it could be that fasting of overweight mice triggers less extreme responses because of the presence of larger reserves. Taken together, the survival and kidney function data as well as the gene expression findings suggest that fasting has a more robust effect in young mice, although in aged mice it is still sufficient to induce a protective effect against IRI.

Although extended criteria donors are getting more and more accepted as an alternative for optimal donors, the outcome after transplantation of an ECD kidney is worse, with higher risks of DGF, acute rejection and graft failure [25]. Since age is a factor that cannot be influenced, reducing the consequences of ischemia is all the more warranted. To elucidate the mechanisms responsible for the protective effect induced by fasting, we performed microarray analysis after fasting. Our analysis revealed several interesting pathways regulated in kidneys from both young-lean and aged-overweight male mice, thereby being independent of age, weight and genetic background. After clustering of the pathways according to their known or predicted biological function, three biological processes stood out, namely: retinol biosynthesis, fatty acid oxidation and the stress response pathway Nrf2 (see Tables 1 and2). Up-regulation of the retinoid X receptors (RXRs) together with retinol biosynthesis was responsible for over a quarter of all overrepresented pathways. RXRs are able to bind with multiple factors like the peroxisome proliferator-activated receptors (PPARs). Depending on the different complexes made, RXRs are involved in many processes. They are able to reduce inflammatory processes and contribute to the reduction of the acute phase response, an inflammatory response occurring after invasive surgery resembling renal IRI [26]. Activation of RXR also stimulates fatty acid oxidation and transport and is thus partially responsible for the enrichment in fatty acid pathways found after short-term fasting leading to reduced fat synthesis and storage and increased oxidation in order to burn fat for the production of energy as part of a redesign of metabolic processes upon lack of food [27]. Nrf2 and genes involved in the Nrf2-pathway were found to be significantly up-regulated in kidneys of both young and aged mice after 3 days of fasting, although less pronounced in the aged cohort. Nrf2, nuclear factor-erythroid 2 p-45 related factor 2, is known to have both anti-oxidant and anti-inflammatory features and, more specific to IRI, a deficiency of Nrf2 leads to aggravation of ischemic injury in mice [28]–[30]. Previously, we showed that a downstream product of Nrf2, heme oxygenase 1 (HO-1), is markedly increased by 3-day fasting in young mice [9]. Together with the pronounced Nrf2-mediated oxidative stress response pathway found in the current analysis, Nrf2 is likely involved in the stress resistance phenotype after DR in both young-lean and aged-overweight mice. It was recently shown that a Nrf2 activator, CDDO-imidazolide, is able to improve survival after a renal IRI model in mice, inducing similar outcome found with fasting in our mouse model [31]. Whether Nrf2 induction would lead to the same beneficial effects induced by DR and would be safe and feasibly in the clinic, is to be elucidated.

The up-regulation of pathways involved in stress resistance suggests that DR induces a protective state. In addition, we previously showed in young animals that this protection was associated with decreased levels of oxidative injury, cytokine production and inflammation and at six hours after IRI, levels of the pro-inflammatory cytokine interleukin 6 (IL-6) were significantly lower in DR compared to ad libitum fed mice [9], [24]. These early effects support the concept that DR preconditions a protective stress response resulting in a better recovery rather than a faster recovery per se.

In summary, we have shown that fasting induces a robust protection against renal IRI in aged-overweight F1-FVB/C57BL6-hybrid mice as it does in young-lean C57BL/6 mice. These findings suggest a general protective response induced by DR against IRI regardless of age, gender, body weight and genetic background. Gene expression profiles of kidneys of both young and aged mice after fasting show the involvement of retinol biosynthesis as well as stress response pathways. Whether these effects are cell-autonomous or influenced by systemic factors remains unknown and needs further clarification. Finally, contrary to common believe short-term preoperative dietary restriction rather than hyper alimentation improves stress resistance and recovery from surgically induced renal IRI. Therefore, translation of preoperative dietary restriction to the clinic seems to be the next logical step, whereby older and overweight patients do not have to be excluded from its benefits.

## Experimental Procedures

### Ethics statement

All animal experiments are done according to the Dutch National Experiments on Animal Act via the EU adopted Directive 86/609/EEC (1986) and had the approval of the local Animal Experiments Committee of the National Institute of Public Health and the Environment (RIVM), the Netherlands (protocol number: 201100308). To ameliorate suffering of the animals, the mice were given 0.5 ml PBS supplemented with 2.4 µg buprenorphine after surgery in order to compensate for fluid loss and provide analgesia and an identical analgesic dose of buprenorphine was administered in the morning after surgery. The method of sacrifice was exsanguination, done under anesthesia by intramuscular injection of a Ketamine–Rompun mixture.

### Animals

All aged mice were bred, raised and kept under identical SPF conditions as described [32]. Male (n = 16) and female (n = 22) wild type FVB-C57BL/6J F1-hybrid mice, with an average age of 72 and 74 weeks and average weight of 47.1 and 47.4 grams, respectively, were used. Animals were kept under standard laboratory conditions (temperature ±20°C, 12 hour light/12 hour dark) with either 1 animal (male) or 2 animals (females) per cage and were allowed free access to water and food (Special Diet Services, UK) unless noted otherwise. The conditions of and procedures done in the young male C57BL/6J mice are described elsewhere [9].

### Preoperative fasting

Mice were pair matched based on body weight and sex. Subsequently, the animals were randomly assigned to one of two groups. The first group had unlimited access to food and water preoperatively. The average amount of food eaten in the ad libitum fed male and female group was approximately 5.7 and 4.4 grams/day respectively. The second group was fasted for three days preoperatively with no access to food and unlimited access to water. Fasted animals did not show any morbidity due to the fasting. Directly after surgery, all mice had unlimited access to food and water. For the microarray analysis, 10 other male mice were also assigned to either ad libitum food or fasting for three days, after which the mice were sacrificed.

### Surgical procedure

Renal IRI was performed as described previously [9], [14]. In brief, mice were anaesthetized by isoflurane inhalation (5% isoflurane initially and then 2–2.5% with oxygen for maintenance). A midline abdominal incision was performed and the renal artery and vein of both kidneys were exposed. In the male mice, the pedicles of both kidneys were occluded for 37 minutes. As female mice are more resistant to ischemic renal damage [33], [34], the ischemic time in female mice was extended to 60 minutes. Purple discoloration of the kidneys confirmed ischemia macroscopically. After removal of the clamps, reperfusion was confirmed when the kidney color turned back to normal. The incision was closed in two layers with 5/0 sutures. After closure, the animals were subcutaneously injected with 0.5 ml PBS supplemented with 2.4 µg buprenorphine in order to compensate for fluid loss and provide analgesia. An identical analgesic dose of buprenorphine was administered in the morning after surgery to ameliorate suffering. The mice used for microarray analysis did not undergo renal IRI, instead were sacrificed right after the dietary intervention.

### Follow-up and serum measurements

The animals were followed up to 28 days postoperatively unless they died or were sacrificed as a result of morbidity (ruffled fur, decreased mobility, cold to the touch, excessive weight loss), or noted otherwise. During follow-up, they were inspected at least daily. Body weight was measured daily in the first postoperative week and on day 10, 14, 17, 21 and 28 if applicable. At day 28, all remaining animals were euthanized after which the abdominal cavity was opened for inspection of the kidneys. A blood sample of 50 µl was collected by orbital puncture while the animals were anaesthetized as well as on postoperative day 1, 2, 3, 7 and 28 if applicable. Serum urea and creatinine levels were measured using the QuantiChrom assay kits (DIUR-500 and DICt-500, Gentaur Europe, Brussels, Belgium). Statistical analysis was performed with IBM SPSS statistics version 20.0, GraphPad Prism version 5.0, R software and/or Microsoft Excel. Statistical significance was defined as p-value <0.05 unless noted otherwise. For assessment of the kidney function, the one-way ANOVA was used including the Bonferroni test for multiple comparisons. Means were compared using either the non-parametric Mann-Whitney U test or the independent t-test for parametric data. The survival curves were compared using the Log-rank (Mantel-Cox) test and visualized by the Kaplan-Meier curve.

### Histology

After euthanasia, both kidneys were harvested. Half of the left and the right kidney of each mouse were fixed in formalin for histological analysis in Hematoxylin and Eosin (HE) stained sections. Kidneys were analyzed in a blinded manner. Semi quantitative scoring of the severity of the lesions was performed for acute tubular necrosis (0–5) and tubular epithelial regeneration (0–4). Scores were defined as: 0 =  none; 1 = minimal; 2 = mild; 3 = moderate; 4 = marked and, if applicable, 5 = severe necrosis or regeneration. Pathology scores were analyzed with the Kruskal-Wallis test for K independent samples and the Dunn's test for multiple comparisons.

### Microarray analysis

Of the 10 male mice used for microarray analysis, half of both the left and the right kidney (taken in longitudinal direction) were snap frozen in liquid nitrogen until further analysis directly after fasting. For gene expression analysis, total RNA was extracted from frozen kidney tissue using the QIAzol lysis Reagent and miRNAeasy Mini Kit (QIAGEN, Hilden, Germany), as described in the Qiagen protocol (males: fasted n = 5, ad libitum n = 5). Addition of wash buffers RPE and RWT (QIAGEN) was done mechanically by using the QIAcube (QIAGEN, Hilden, Germany) via the miRNeasy program. RNA was eluted in RNase free water and stored at −80°C. The concentration of RNA was measured by Nanodrop (Thermo Scientific). Quality assessment of the RNA was done using the 2100 Bio-Analyzer (Agilent Technologies, Amstelveen, the Netherlands). The quality of the RNA is expressed as the RNA integrity number (RIN, range 0–10). Samples with a RIN below 8 were excluded from analysis. RNA was further processed for hybridization to Affymetrix HT MG-430 PM Array Plates at the Microarray Department of the University of Amsterdam, the Netherlands according to Affymetrix protocols. Four to six biological replicates were used for each group. Quality control and normalization were performed using the pipeline at the www.arrayanalysis.org website (Maastricht University, the Netherlands). Normalization was done via the Robust Multichip Average (RMA) algorithm. Normalization output consisted of data for 45141 probe sets, with several probe sets corresponding to the same Entrez Gene ID. Complete raw and normalized microarray data and their MIAME compliant metadata have been deposited at GEO (www.ncbi.nlm.nih.gov/geo) under accession number GSE52982. Gene expression data were compared using ANOVA with correction for multiple testing using the false discovery rate (FDR) according to Benjamini and Hochberg [35]. Fold changes are expressed as the geometric mean per fasted group against the corresponding ad libitum group. Cutoff values for a significant difference were put at FDR <5%. Functional annotation and overrepresentation analyses were performed with Ingenuity software (http://www.ingenuity.com/products/ipa). For comparison analysis with young male mice, the same procedure was performed in kidney tissue of male C57BL/6J mice (fasted n = 4, ad libitum fed n = 5). For the analyses done in the genes overlapping between aged-overweight and young-lean male mice, the mean fold ratios and geometric mean p-values of both groups were used. No female mice were available for the microarray analysis in young and aged mice.

## Supporting Information

File S1 **Combined file of supporting tables.** **Table S1**: Top genes up-regulated in aged mice fasted for 3 days. Top gene lists of up-regulated genes in aged-overweight mice fasted for 3 days, with corresponding symbols, log fold ratios and p-values. All genes with a fold change >5 (log fold ratio (−)1.609) are listed. **Table S2**: Top genes down-regulated in aged mice fasted for 3 days. Top gene lists of down-regulated genes in aged-overweight mice fasted for 3 days, with corresponding symbols, log fold ratios and p-values. All genes with a fold change >5 (log fold ratio (−)1.609) are listed. **Table S3**: Top genes up-regulated in young mice fasted for 3 days. Top gene lists of up-regulated genes in young-lean mice fasted for 3 days, with corresponding symbols, log fold ratios and p-values. All genes with a fold change >5 (log fold ratio (−)1.609) are listed. **Table S4**: Top genes down-regulated in young mice fasted for 3 days. Top gene lists of down-regulated genes in young-lean mice fasted for 3 days, with corresponding symbols, log fold ratios and p-values. All genes with a fold change >5 (log fold ratio (−)1.609) are listed.
(ZIP)
