# Supplementary material for: Preoperative Fasting Protects against Renal Ischemia-Reperfusion Injury in Aged and Overweight Mice
Source: PLoS One. 2014 Jun 24;9(6):e100853. doi: 10.1371/journal.pone.0100853 (PMC4069161; doi:10.1371/journal.pone.0100853)
Supplement: File S1 — Combined file of supporting tables. Table S1: Top genes up-regulated in aged mice fasted for 3 days. Top gene lists of up-regulated genes in aged-overweight mice fasted for 3 days, with corresponding symbols, log fold ratios and p-values. All genes with a fold change >5 (log fold ratio (−)1.609) are listed. Table S2: Top genes down-regulated in aged mice fasted for 3 days. Top gene lists of down-regulated genes in aged-overweight mice fasted for 3 days, with corresponding symbols, log fold ratios and p-values. All genes with a fold change >5 (log fold ratio (−)1.609) are listed. Table S3: Top genes up-regulated in young mice fasted for 3 days. Top gene lists of up-regulated genes in young-lean mice fasted for 3 days, with corresponding symbols, log fold ratios and p-values. All genes with a fold change >5 (log fold ratio (−)1.609) are listed. Table S4: Top genes down-regulated in young mice fasted for 3 days. Top gene lists of down-regulated genes in young-lean mice fasted for 3 days, with corresponding symbols, log fold ratios and p-values. All genes with a fold change >5 (log fold ratio (−)1.609) are listed. (ZIP) [file pone.0100853.s001.zip › Table S4.docx]

**Table S4. Top genes down-regulated in young mice fasted for 3 days**

| **Genes YOUNG down-regulated** | **Symbol** | **Log FR** | **P-value** |
| --- | --- | --- | --- |
| Solute carrier family 22, member 7 | SLC22A7 | -3.713 | 8.28E-08 |
| Kinesin family member 20B | KIF20B | -3.379 | 4.11E-06 |
| Hydroxy-delta-5-steroid dehydrogenase 3, beta- and steroid-isomerase 1 | HSD3B1 | -2.950 | 1.04E-06 |
| Branched chain amino-acid transaminase 1 | BCAT1 | -2.838 | 1.94E-05 |
| Histone cluster 2, H3c | HIST2H3C | -2.818 | 5.05E-07 |
| major facilitator superfamily domain containing 2A | MFSD2A | -2.756 | 1.27E-04 |
| isopentenyl-diphosphate delta isomerase 1 | IDI1 | -2.712 | 2.96E-08 |
| solute carrier family 9, subfamily A, member 8 | SLC9A8 | -2.679 | 1.07E-07 |
| gamma-aminobutyric acid (GABA) A receptor, beta 3 | GABRB3 | -2.502 | 1.25E-05 |
| solute carrier family 7, member 13 | SLC7A13 | -2.418 | 3.09E-02 |
| heat shock transcription factor 2 binding protein | HSF2BP | -2.337 | 7.43E-07 |
| myosin VA (heavy chain 12, myoxin) | MYO5A | -2.334 | 5.95E-06 |
| synapsin III | SYN3 | -2.324 | 4.15E-06 |
| collagen, type III, alpha 1 | COL3A1 | -2.229 | 1.05E-06 |
| ST8 alpha-N-acetyl-neuraminide alpha-2,8-sialyltransferase 1 | ST8SIA1 | -2.212 | 7.42E-07 |
| chemokine ligand 9 | CXCL9 | -2.184 | 1.17E-04 |
| ornithine decarboxylase 1 | ODC1 | -2.106 | 4.25E-07 |
| cytochrome P450, family 51, subfamily A, polypeptide 1 | CYP51A1 | -2.088 | 2.82E-07 |
| family with sequence similarity 151, member A | FAM151A | -2.018 | 5.95E-06 |
| GRB10 interacting GYF protein 2 | GIGYF2 | -1.968 | 6.45E-06 |
| aldo-keto reductase family 1, member C3 | AKR1C3 | -1.943 | 1.11E-04 |
| C1q and tumor necrosis factor related protein 3 | C1QTNF3 | -1.943 | 1.60E-06 |
| midkine (neurite growth-promoting factor 2) | MDK | -1.937 | 6.72E-05 |
| kinesin family member 12 | KIF12 | -1.928 | 8.78E-08 |
| SLIT and NTRK-like family, member 6 | SLITRK6 | -1.890 | 9.74E-05 |
| McKusick-Kaufman syndrome | MKKS | -1.843 | 2.30E-05 |
| 3-hydroxy-3-methylglutaryl-CoA reductase | HMGCR | -1.838 | 7.06E-06 |
| solute carrier family 35, member F1 | SLC35F1 | -1.809 | 6.67E-07 |
| solute carrier family 34, member 3 | SLC34A3 | -1.782 | 3.50E-05 |
| C-type lectin domain family 2, member h | Clec2h | -1.780 | 7.84E-05 |
| guanylate binding protein 6 | Gbp6 | -1.778 | 1.10E-04 |
| solute carrier family 8, member 1 | SLC8A1 | -1.770 | 6.25E-06 |
| UDP-Gal:beta GlcNAc beta 1,4- galactosyltransferase, polypeptide 5 | B4GALT5 | -1.755 | 6.58E-07 |
| protein Z, vitamin K-dependent plasma glycoprotein | PROZ | -1.745 | 9.18E-07 |
| centromere protein J | CENPJ | -1.737 | 1.24E-06 |
| histone cluster 1, H2ab | Hist1h2ab | -1.708 | 5.22E-05 |
| UDP glucuronosyltransferase 2 family, polypeptide B15 | UGT2B15 | -1.680 | 6.53E-04 |
| annexin A13 | ANXA13 | -1.678 | 1.23E-04 |
| glycerate kinase | GLYCTK | -1.611 | 7.42E-07 |

**Table S4.** Top gene lists of down-regulated genes in young-lean mice fasted for 3 days, with corresponding
symbols, log fold ratios and p-values. All genes with a fold change >5 (log fold ratio (-)1.609) are listed.
