# Supplementary material for: Preoperative Fasting Protects against Renal Ischemia-Reperfusion Injury in Aged and Overweight Mice
Source: PLoS One. 2014 Jun 24;9(6):e100853. doi: 10.1371/journal.pone.0100853 (PMC4069161; doi:10.1371/journal.pone.0100853)
Supplement: File S1 — Combined file of supporting tables. Table S1: Top genes up-regulated in aged mice fasted for 3 days. Top gene lists of up-regulated genes in aged-overweight mice fasted for 3 days, with corresponding symbols, log fold ratios and p-values. All genes with a fold change >5 (log fold ratio (−)1.609) are listed. Table S2: Top genes down-regulated in aged mice fasted for 3 days. Top gene lists of down-regulated genes in aged-overweight mice fasted for 3 days, with corresponding symbols, log fold ratios and p-values. All genes with a fold change >5 (log fold ratio (−)1.609) are listed. Table S3: Top genes up-regulated in young mice fasted for 3 days. Top gene lists of up-regulated genes in young-lean mice fasted for 3 days, with corresponding symbols, log fold ratios and p-values. All genes with a fold change >5 (log fold ratio (−)1.609) are listed. Table S4: Top genes down-regulated in young mice fasted for 3 days. Top gene lists of down-regulated genes in young-lean mice fasted for 3 days, with corresponding symbols, log fold ratios and p-values. All genes with a fold change >5 (log fold ratio (−)1.609) are listed. (ZIP) [file pone.0100853.s001.zip › Table S1.docx]

**Table S1. Top genes up-regulated in aged mice fasted for 3 days**

| **Genes AGED up-regulated** | **Symbol** | **Log FR** | **P-value** |
| --- | --- | --- | --- |
| Cytochrome P450, family 4, subfamily a, polypeptide 14 | Cyp4a14 | 5.842 | 5.46E-06 |
| 3-hydroxy-3-methylglutaryl-CoA synthase 2 | HMGCS2 | 5.125 | 1.48E-06 |
| Phosphoenolpyruvate carboxykinase 1 | PCK1 | 3.260 | 1.83E-04 |
| Acyl-CoA thioesterase 1 | ACOT1 | 2.609 | 6.18E-06 |
| Pyruvate dehydrogenase kinase, isozyme 4 | PDK4 | 2.508 | 2.44E-03 |
| Solute carrier family 38, member 3 | SLC38A3 | 2.365 | 5.57E-05 |
| Cytochrome P450, family 4, subfamily a, polypeptide 11 | Cyp4a11 | 2.250 | 2.15E-05 |
| Mitochondrial amidoxime reducing component 1 | MARC1 | 2.209 | 6.23E-03 |
| WD repeat and SOCS box containing 1 | WSB1 | 2.020 | 3.64E-06 |
| Inhibitor of DNA binding 1 | ID1 | 1.937 | 6.18E-06 |
| Kv channel-interacting protein 2 | Kcnip2 | 1.913 | 6.50E-06 |
| inhibitor of DNA binding 3 | ID3 | 1.903 | 7.35E-06 |
| acyl-CoA thioesterase 1 | Acot1 | 1.902 | 4.94E-06 |
| betaine--homocysteine S-methyltransferase | BHMT | 1.890 | 2.60E-02 |
| insulin-like growth factor binding protein 1 | IGFBP1 | 1.860 | 8.55E-03 |
| group-specific component (vitamin D binding protein) | GC | 1.853 | 3.60E-03 |
| apolipoprotein D | APOD | 1.808 | 3.82E-03 |
| fibrinogen alpha chain | FGA | 1.802 | 9.00E-04 |
| microsomal glutathione S-transferase 1 | MGST1 | 1.792 | 6.30E-06 |
| nuclear factor, erythroid 2-like 2 | NFE2L2 | 1.767 | 4.56E-03 |
| fibrinogen gamma chain | FGG | 1.766 | 1.29E-02 |
| complement component 3 | C3 | 1.756 | 1.18E-04 |
| aldehyde dehydrogenase family 1, subfamily A7 | Aldh1a7 | 1.722 | 2.02E-06 |
| glutathione S-transferase alpha 5 | GSTA5 | 1.708 | 1.63E-05 |
| amylase, alpha 1A (salivary) | AMY1A | 1.706 | 1.16E-03 |
| acyl-CoA thioesterase 2 | ACOT2 | 1.705 | 1.48E-06 |
| ATP-binding cassette, sub-family B (MDR/TAP), member 1B | Abcb1b | 1.662 | 5.84E-06 |
| aldehyde dehydrogenase 1 family, member A1 | ALDH1A1 | 1.655 | 4.12E-05 |
| arginase 2 | ARG2 | 1.641 | 8.57E-05 |
| cytochrome c oxidase subunit VIb polypeptide 2 (testis) | COX6B2 | 1.624 | 1.98E-06 |

**Table S1.** Top gene lists of up-regulated genes in aged-overweight mice fasted for 3 days, with
corresponding symbols, log fold ratios and p-values. All genes with a fold change >5
(log fold ratio (-)1.609) are listed.
