# Supplementary material for: Preoperative Fasting Protects against Renal Ischemia-Reperfusion Injury in Aged and Overweight Mice
Source: PLoS One. 2014 Jun 24;9(6):e100853. doi: 10.1371/journal.pone.0100853 (PMC4069161; doi:10.1371/journal.pone.0100853)
Supplement: File S1 — Combined file of supporting tables. Table S1: Top genes up-regulated in aged mice fasted for 3 days. Top gene lists of up-regulated genes in aged-overweight mice fasted for 3 days, with corresponding symbols, log fold ratios and p-values. All genes with a fold change >5 (log fold ratio (−)1.609) are listed. Table S2: Top genes down-regulated in aged mice fasted for 3 days. Top gene lists of down-regulated genes in aged-overweight mice fasted for 3 days, with corresponding symbols, log fold ratios and p-values. All genes with a fold change >5 (log fold ratio (−)1.609) are listed. Table S3: Top genes up-regulated in young mice fasted for 3 days. Top gene lists of up-regulated genes in young-lean mice fasted for 3 days, with corresponding symbols, log fold ratios and p-values. All genes with a fold change >5 (log fold ratio (−)1.609) are listed. Table S4: Top genes down-regulated in young mice fasted for 3 days. Top gene lists of down-regulated genes in young-lean mice fasted for 3 days, with corresponding symbols, log fold ratios and p-values. All genes with a fold change >5 (log fold ratio (−)1.609) are listed. (ZIP) [file pone.0100853.s001.zip › Table S2.docx]

**Table S2. Top genes down-regulated in aged mice fasted for 3 days**

| **Genes AGED down-regulated** | **Symbol** | **Log FR** | **P-value** |
| --- | --- | --- | --- |
| Solute carrier family 22, member 7 | SLC22A7 | -2.670 | 7.74e-05 |
| Major facilitator superfamily domain containing 2A | MFSD2A | -2.357 | 2.03e-04 |
| Solute carrier family 8, member 1 | SLC8A1 | -2.302 | 1.76e-04 |
| Antisense Igf2r RNA | Airn | -2.097 | 1.17e-05 |
| Midkine | MDK | -1.850 | 2.84e-04 |
| Branched chain amino-acid transaminase 1 | BCAT1 | -1.686 | 1.64e-03 |
| Carbonix anhydrase IV | CA4 | -1.680 | 2.62e-06 |
| Isopentenyl-disphosphate delta isomerase 1 | IDI1 | -1.674 | 2.25e-05 |
| Solute carrier family 9, subfamily A, member 8 | SLC9A8 | -1.647 | 1.29e-06 |
| Ring finger protein 183 | RNF183 | -1.606 | 6.58e-05 |

**Table S2.** Top gene lists of down-regulated genes in aged-overweight mice fasted for 3 days,
with corresponding symbols, log fold ratios and p-values. All genes with a fold change >5
(log fold ratio (-)1.609) are listed.
