# Supplementary material for: Preoperative Fasting Protects against Renal Ischemia-Reperfusion Injury in Aged and Overweight Mice
Source: PLoS One. 2014 Jun 24;9(6):e100853. doi: 10.1371/journal.pone.0100853 (PMC4069161; doi:10.1371/journal.pone.0100853)
Supplement: File S1 — Combined file of supporting tables. Table S1: Top genes up-regulated in aged mice fasted for 3 days. Top gene lists of up-regulated genes in aged-overweight mice fasted for 3 days, with corresponding symbols, log fold ratios and p-values. All genes with a fold change >5 (log fold ratio (−)1.609) are listed. Table S2: Top genes down-regulated in aged mice fasted for 3 days. Top gene lists of down-regulated genes in aged-overweight mice fasted for 3 days, with corresponding symbols, log fold ratios and p-values. All genes with a fold change >5 (log fold ratio (−)1.609) are listed. Table S3: Top genes up-regulated in young mice fasted for 3 days. Top gene lists of up-regulated genes in young-lean mice fasted for 3 days, with corresponding symbols, log fold ratios and p-values. All genes with a fold change >5 (log fold ratio (−)1.609) are listed. Table S4: Top genes down-regulated in young mice fasted for 3 days. Top gene lists of down-regulated genes in young-lean mice fasted for 3 days, with corresponding symbols, log fold ratios and p-values. All genes with a fold change >5 (log fold ratio (−)1.609) are listed. (ZIP) [file pone.0100853.s001.zip › Table S3.docx]

**Table S3. Top genes up-regulated in young mice fasted for 3 days**

| **Genes YOUNG up-regulated** | **Symbol** | **Log FR** | **P-value** |
| --- | --- | --- | --- |
| 3-hydroxy-3-methylglutaryl-CoA synthase 2 (mitochondrial) | HMGCS2 | 5.227 | 1.69E-07 |
| cytochrome P450, family 4, subfamily a, polypeptide 14 | Cyp4a14 | 4.842 | 9.85E-07 |
| carbonyl reductase 3 | CBR3 | 3.565 | 4.18E-08 |
| aldehyde dehydrogenase 1 family, member A1 | ALDH1A1 | 3.307 | 5.82E-09 |
| pyruvate dehydrogenase kinase, isozyme 4 | PDK4 | 3.071 | 4.25E-07 |
| cell death-inducing DFFA-like effector a | CIDEA | 2.841 | 2.45E-02 |
| receptor accessory protein 6 | REEP6 | 2.757 | 2.82E-07 |
| aldehyde dehydrogenase family 1, subfamily A7 | Aldh1a7 | 2.625 | 2.57E-07 |
| phosphoenolpyruvate carboxykinase 1 (soluble) | PCK1 | 2.608 | 5.70E-05 |
| acyl-CoA thioesterase 1 | ACOT1 | 2.590 | 8.71E-07 |
| arbonyl reductase 1 | CBR1 | 2.573 | 8.78E-08 |
| nuclear factor, erythroid 2-like 2 | NFE2L2 | 2.573 | 2.00E-07 |
| cytochrome P450, family 2, subfamily d, polypeptide 22 | Cyp2d22 | 2.531 | 1.21E-07 |
| fibrinogen gamma chain | FGG | 2.508 | 2.66E-06 |
| solute carrier family 25, member 25 | SLC25A25 | 2.507 | 5.74E-06 |
| atonal homolog 7 (Drosophila) | ATOH7 | 2.478 | 3.35E-08 |
| solute carrier family 38, member 3 | SLC38A3 | 2.456 | 4.25E-07 |
| group-specific component (vitamin D binding protein) | GC | 2.437 | 5.65E-06 |
| monoamine oxidase B | MAOB | 2.363 | 1.67E-05 |
| mitochondrial amidoxime reducing component 1 | MARC1 | 2.270 | 1.94E-05 |
| arginase 2 | ARG2 | 2.180 | 3.60E-06 |
| complement component 3 | C3 | 2.176 | 9.85E-07 |
| glutathione S-transferase alpha 5 | GSTA5 | 2.166 | 3.73E-05 |
| fibrinogen alpha chain | FGA | 2.086 | 2.12E-05 |
| lectin, galactoside-binding, soluble, 4 | LGALS4 | 2.051 | 5.67E-06 |
| insulin-like growth factor binding protein 1 | IGFBP1 | 2.035 | 1.03E-03 |
| CCR4 carbon catabolite repression 4-like (S. cerevisiae) | CCRN4L | 2.006 | 8.54E-05 |
| perilipin 5 | PLIN5 | 1.981 | 1.05E-06 |
| abhydrolase domain containing 15 | ABHD15 | 1.964 | 3.11E-07 |
| paternally expressed 3 | PEG3 | 1.947 | 1.97E-05 |
| retinol dehydrogenase 1 (all trans) | Rdh1 | 1.916 | 4.25E-07 |
| early growth response 1 | EGR1 | 1.901 | 2.54E-02 |
| pyridoxal (pyridoxine, vitamin B6) kinase | PDXK | 1.894 | 1.15E-05 |
| betaine--homocysteine S-methyltransferase | BHMT | 1.886 | 4.91E-03 |
| suprabasin | SBSN | 1.879 | 5.74E-06 |
| cold inducible RNA binding protein | CIRBP | 1.875 | 1.38E-05 |
| regulation of nuclear pre-mRNA domain containing 2 | RPRD2 | 1.850 | 3.04E-06 |
| period circadian clock 1 | PER1 | 1.848 | 4.56E-05 |
| RIKEN cDNA 6030422H21 gene | 6030422H21Rik | 1.847 | 2.93E-03 |
| retinol saturase (all-trans-retinol 13,14-reductase) | RETSAT | 1.825 | 8.77E-07 |
| chromosome 10 open reading frame 10 | C10orf10 | 1.822 | 2.73E-03 |
| inhibitor of DNA binding 1 | ID1 | 1.814 | 4.89E-07 |
| amyotrophic lateral sclerosis 2 (juvenile) chromosome region, candidate 12 | ALS2CR12 | 1.814 | 1.21E-04 |
| cytochrome P450, family 4, subfamily A, polypeptide 11 | CYP4A11 | 1.792 | 3.47E-07 |
| cytochrome c oxidase subunit VIIIb | Cox8b | 1.769 | 4.64E-02 |
| ATP-binding cassette, sub-family C (CFTR/MRP), member 4 | ABCC4 | 1.764 | 1.10E-06 |
| angiopoietin-like 4 | ANGPTL4 | 1.748 | 2.64E-05 |
| zinc finger CCCH-type containing 6 | ZC3H6 | 1.743 | 7.07E-06 |
| solute carrier family 22 (organic anion transporter), member 8 | SLC22A8 | 1.730 | 3.19E-05 |
| Kv channel-interacting protein 2 | Kcnip2 | 1.702 | 5.03E-07 |
| ganglioside-induced differentiation-associated-protein 10 | Gdap10 | 1.690 | 5.67E-06 |
| NAD(P)H dehydrogenase, quinone 1 | NQO1 | 1.688 | 7.41E-07 |
| ATP-binding cassette, sub-family B (MDR/TAP), member 1B | Abcb1b | 1.686 | 3.40E-07 |
| acyl-CoA thioesterase 2 | ACOT2 | 1.679 | 1.55E-05 |
| FBJ murine osteosarcoma viral oncogene homolog | FOS | 1.660 | 4.41E-02 |
| 2-hydroxyacyl-CoA lyase 1 | HACL1 | 1.660 | 5.67E-06 |
| DAZ interacting zinc finger protein 1 | DZIP1 | 1.657 | 1.08E-03 |
| butyrobetaine, 2-oxoglutarate dioxygenase 1 | BBOX1 | 1.656 | 7.42E-07 |
| amylase, alpha 1A | AMY1A | 1.644 | 6.26E-06 |
| ceruloplasmin (ferroxidase) | CP | 1.641 | 4.15E-06 |
| fatty acid binding protein 4, adipocyte | FABP4 | 1.640 | 1.16E-04 |
| G0/G1switch 2 | G0S2 | 1.629 | 1.05E-06 |
| vanin 1 | VNN1 | 1.621 | 4.25E-07 |
| ethanolamine-phosphate phospho-lyase | ETNPPL | 1.617 | 5.58E-06 |
| apolipoprotein D | APOD | 1.617 | 6.81E-04 |

**Table S3**. Top gene lists of up-regulated genes in young-lean mice fasted for 3 days, with corresponding symbols,
log fold ratios and p-values. All genes with a fold change >5 (log fold ratio (-)1.609) are listed.
